# Proteins from the past

**DOI:** 10.7554/eLife.20877

**Published:** 2016-09-27

**Authors:** Adam F Wallace, James D Schiffbauer

**Affiliations:** 1 Department of Geological Sciences, University of Delaware, Newark, United Statesafw@udel.edu; 2 Department of Geological Sciences, University of Missouri, Columbia, United Statesschiffbauerj@missouri.edu

**Keywords:** Struthio camelus, paleoproteomics, eggshell, molecular dynamics, paleontology, biomineralization, Other

## Abstract

Some fragments of ancient protein are less prone to degradation because they bind strongly to the surfaces of minerals.

**Related research article** Demarchi B, Hall S, Roncal-Herrero T, Freeman CL, Woolley J, Crisp MK, Wilson J, Fotakis A, Fischer R, Kessler B, Jersie-Christensen RR, Olsen JV, Haile J, Thomas J, Marean CW, Parkington J, Presslee S, Lee-Thorp J, Ditchfield P, Hamilton JF, Ward MW, Wang CM, Shaw MD, Harrison T, Domínguez-Rodrigo M, MacPhee RDE, Kwekason A, Ecker M, Horwitz LK, Chazan M, Kröger R, Thomas-Oates J, Harding JH, Cappellini E, Penkman K, Collins MJ. 2016. Protein sequences bound to mineral surfaces persist into deep time. *eLife* **5**:e17092. doi: 10.7554/eLife.17092

If you have ever walked along the shore, chances are you have come across a clam or snail shell in the sand. You may have even been compelled to briefly pick it up and take in its natural beauty before casually casting it aside and continuing on your way. You may not have been aware, however, that in that moment you were holding one of nature’s foremost feats of materials engineering, the likes of which remain unparalleled by human endeavors. A shell is an example of a biomineral – a hybrid material in which biologically produced macromolecules are intimately associated with mineral. This combination of inorganic and organic components often results in the emergence of unique and desirable physical properties that are exploited by the organisms that manufacture them for a variety of applications, including structural support, protection and light harvesting (Wallace et al., 2012).

It is not clear exactly how long organisms have been able to actively direct the deposition of minerals to their advantage. The fossil record indicates that controlled biomineralization in animals started ca. 551 million years ago, during the last 10 million years of the Precambrian (Grant, 1990; Grotzinger et al., 2000; Hua et al., 2007). By the end of the ‘Cambrian Explosion’, some 35 million years later, biomineralization had largely spread through the tree of animal life (Bengtson and Conway Morris, 1992). Indeed, biomineralized skeletal materials appeared within many newly evolved clades, possibly as a result of changes to the ecological landscape that favored species with good support and defense structures (Schiffbauer et al., 2016). Prior to this, the Earth’s biota had consisted primarily of either single-celled or soft-bodied organisms whose preservation in the fossil record probably involved a variety of microbe-mediated processes, including mineral deposition following death (Laflamme et al., 2011; Nutman et al., 2016; Schiffbauer et al., 2014; Wacey et al., 2014). Taken together, these two types of biominerals (one produced by microbe-mediated processes, the other produced by biologically directed deposition) comprise the bulk of the record of life on Earth, and are the primary sources of biological, ecological and environmental information extending deep into geologic time.

The record of controlled biomineralization has been heavily scrutinized to reveal evolutionary trends and variations in ecological pressures, such as predator-prey interactions (Huntley and Kowalewski, 2007) and parasite-host interactions (Huntley and De Baets, 2015) through time. In addition, trace element and isotope signatures within these materials have been used to infer past environmental conditions (Dalbeck et al., 2011). The record of soft tissues, on the other hand, has received far less attention, because organic materials are much more likely to degrade over time. However, discoveries in recent years – such as the observation of what might be collagenous material in dinosaur bones (Schweitzer et al., 2005) – have indicated that the longevity of some mineral-associated organic material is much greater than expected. There is, therefore, a clear need to understand the mechanisms through which biological materials may be stabilized at the molecular level for extended, geologically significant periods of time (Schweitzer et al., 2013).

Now, in *eLife*, Beatrice Demarchi and Matthew Collins of the University of York and co-workers – including researchers from Canada, Denmark, Israel, South Africa, Spain, Tanzania, the United Kingdom and the Unites States – report that calcium carbonate (calcite) has an important role in retarding the degradation of protein fragments within ostrich eggshells (Demarchi et al., 2016). Demarchi et al. started by extracting and characterizing proteinaceous material associated with ostrich eggshells from several archaeological and paleontological sites in Africa. A comparison of the peptides extracted from the samples revealed that, despite the samples having a range of different ages and thermal histories, remnants of two eggshell proteins (SCA-1 and SCA-2) remained intact. Although the abundance of recoverable protein declined as a function of sample age, the vast majority of peptides obtained from samples aged between 150,000 and 3.8 million years could be attributed to a domain of SCA-1 that is rich in aspartic acid. The consistency of this finding across multiple sites of differing age verifies that the recovered protein fragments are not derived from exogenous sources but are indeed native components of the eggshell material.

The long-term survival of proteinaceous material in the fossil record provides empirical evidence for highly effective protein stabilization mechanisms. However, when comparing the relative condition of such samples, it is necessary to take into account processes that will cause organic materials to degrade at different rates. Variations in altitude, burial depth, climate and seasonality all have an influence on the local temperature and hence on the rate of chemical reactions that would act to degrade the organic components of biominerals. To account for these effects and place all their samples on the same timeline, Demarchi et al. calculated effective thermal ages for all 24 of their samples. This analysis yielded thermal ages up to ~16 million years, which is approximately 50 times greater than similar calculations have yielded for any previously authenticated protein sequence.

Demarchi et al. then performed molecular dynamics simulations to investigate the potential mechanisms by which calcite surfaces might act to stabilize eggshell proteins. They simulated the association of whole proteins with the mineral surface, and observed that certain domains within these proteins exhibit a high affinity for calcite (Figure 1). Further analysis indicated that the domain in SCA-1 that is rich in aspartic acid (and is also abundant in the ancient eggshell material) binds more tightly to the calcite surface than any other domain they examined. The simulations also indicated that water molecules that are associated with surface-bound peptides are not as dynamic as those in bulk water. On this basis they suggest that proteins associated with calcite or another mineral may be less susceptible to hydrolytic attack by water.

**Figure 1.**
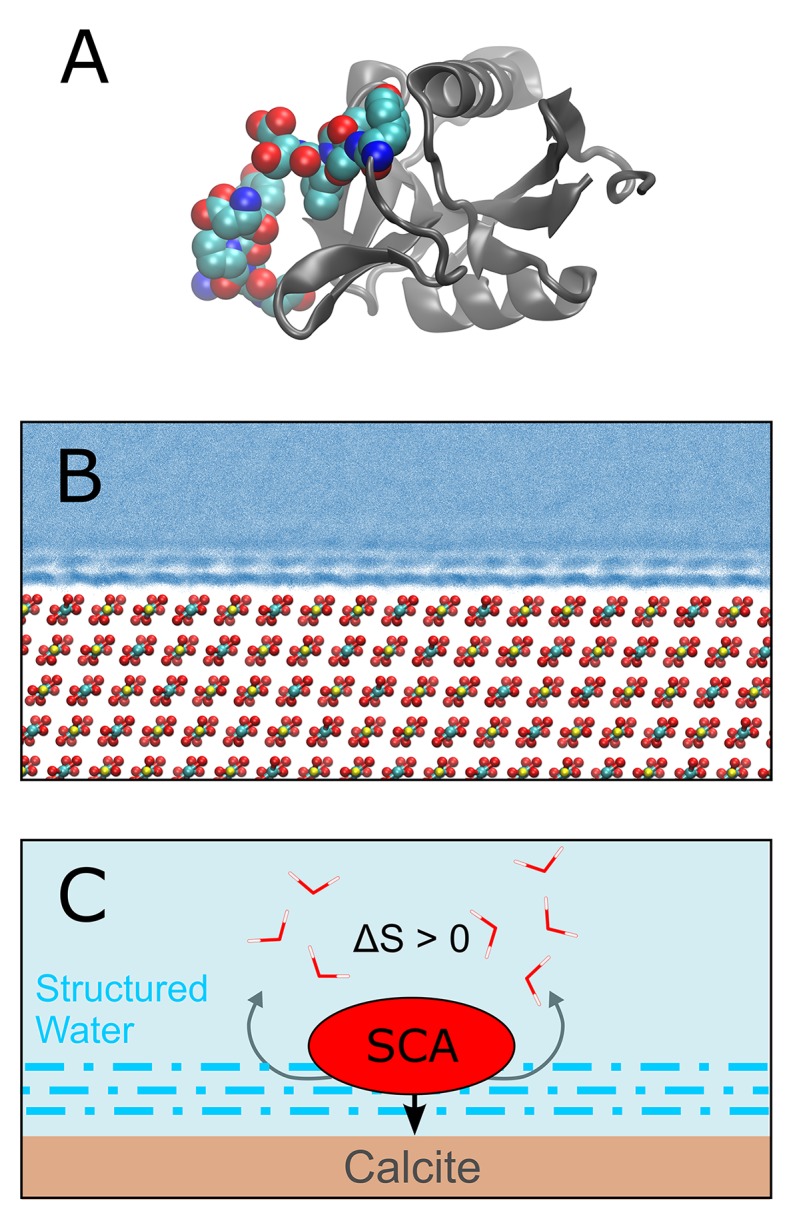
Protein-mineral binding in the presence of water. (**A**) The structure of SCA-1 (struthiocalcin-1): the domain that binds to mineral surfaces is rich in aspartic acid and is shown in color. (**B**) Molecular dynamics simulations performed by one of the present authors (AFW) predict that water molecules adjacent to the surface of calcite (CaCO_3_; Ca = yellow, C = cyan, O = red) form ordered layers. At least three layers of ordered water can be seen in this plot, where each blue point represents the position of a water oxygen during the simulation. Darker blues indicate regions where water is most likely to be. (**C**) Cartoon depicting a protein (SCA) binding to a calcite surface. By disrupting the ordered layers of water molecules (dashed blue lines) near the calcite surface, it is thought that the binding process leads to an increase in entropy (∆S > 0) which, in turn, leads to a more negative binding energy (that is, stronger binding). Demarchi et al. also hypothesize that the water molecules that remain in the ordered layers adjacent to the bound protein are prevented from participating in hydrolysis reactions that would otherwise degrade the protein.

The history of life and environmental change on Earth has long been investigated through the lens of biomineralization. The resolution of the record ultimately depends upon the balance of opposing forces that act to either degrade or preserve biological information. Although the picture that emerges is intrinsically biased, to some degree it may be sharpened if the processes that affect preservation can be better understood. Mechanistic studies in the vein of Demarchi et al. are important milestones en route to this objective, and continued research along these lines will undoubtedly advance our understanding of the process that led to the preferential preservation of biomolecules in the fossil record.
